# The Diagnosis of Syphilitic Skin Affections

**Published:** 1898-01-29

**Authors:** C. F. Marshall

**Affiliations:** Resident Medical Officer, London Lock Hospital


### MEDICAL PROGRESS AND HOSPITAL CLINICS.

[*The Editor will be glad to receive offers of co-operation and contributions from members of the profession. All letters should be addressed to* THE EDITOR, AT THE OFFICE, 28 & 29, SOUTHAMPTON STREET, STRAND, LONDON, W.C.]

### THE DIAGNOSIS OF SYPHILITIC SKIN AFFECTIONS.

By C. F. MARSHALL, M.D., F.R.C.S., Resident Medical Officer, London Lock Hospital.

The lesions of the skin met with in syphilis are often of easy diagnosis, but sometimes cases occur which are very difficult, in the absence of other confirmatory lesions. In this article I propose to point out the main characteristics of syphilitic skin diseases, and afterwards to consider the chief skin diseases which are likely to be mistaken for syphilis, and, *vice versâ*, syphilitic diseases which are likely to be regarded as simple skin affections.

First of all, let us criticise the time-honoured rules applying to syphilitic skin disease, and see if they all hold good.

**Polymorphism.**—This is true in at least 90 per cent. of all cases; that is to say, in most cases of syphilis of the skin more than one type of skin lesion is present, the commonest combinations being the macular, squamous, and papular, any two of these, and often all three, being frequently present at the same time. In fact, this is so often the case that it is usually unwise to diagnose a skin disease presenting only one type of skin lesion as syphilitic unless other typical lesions are present.

**Pigmentation.**—The large majority of skin lesions in syphilis become after a short time pigmented, the tint being usually compared to raw ham; this is no doubt the nearest simile, although many are more of the colour of cooked ham.

**Distribution.**—In secondary syphilis, one of the main characteristics is the distribution of the lesions symmetrically, and this applies especially to the skin. Tertiary lesions are also often more or less symmetrical, but not so much so as secondary ones. The statement that secondary lesions nearly always affect the flexor surfaces of the limbs does not hold good in my experience. I have seen the extensor surfaces affected nearly as often as the flexor.

**The Absence of Itching.**—Although this is one of the main characteristics of syphilis of the skin, it is by no means constant. In fact, it would appear to depend to a great extent on the degree of irritability of the skin of the persons affected.

Now let us take the main types of skin lesions, and compare the non-syphilitic with the syphilitic.

**Erythematous Eruptions.**—The earliest eruption in secondary syphilis is the roseola rash, which varies from a diffuse mottling of the skin to discrete spots, being at first rose colour, but soon fading to greyish-brown. It appears on the chest, abdomen, loins, and thighs. Diagnosis: This is seldom difficult, even apart from the history of a primary sore. Measles and German measles are much darker red and more discrete, and are accompanied by more constitutional disturbance than we usually get in syphilis. Moreover, the concomitant symptoms in these cases are usually conclusive. The only doubtful case I have ever seen (out of many hundreds) was one in which the rash resembled that of scarlet fever for two or three days, being bright red and punctiform, and situated on the chest, abdomen, and thighs, and also accompanied by slight fever. In this case the eruption soon developed into one of syphilitic miliaria.

**Papular Eruptions.**—The papular eruptions of syphilis are usually mixed with the squamous forming the common papulo-squamous syphilide. The miliary and lichenoid syphilides come under this group, but they are very rare apart from other more typical syphilitic lesions. Diagnosis: This depends on the usual associations with other more typical syphilitic lesions; apart from these a papular skin eruption should never be diagnosed as syphilitic.

**Squamous Eruptions.**—These, as stated above, are usually mixed with a papular or macular eruption. Diagnosis: The diagnosis from simple psoriasis is usually fairly easy. In the simple eruption the parts usually affected are the elbows and knees; also the scales are pearly-white and abundant, and when removed have a bright red patch. In syphilitic psoriasis (or the squamous syphilide) the patches are more scattered over the body, do not specially affect the knees and elbows, are of a darker and more dirty colour, have less scales, and the scales are less adherent and hence more easily removed, and leave brownish-red stains.

**Circinate or Annular Eruptions.**—This is a variety of the squamous syphilide, and is usually a late secondary phenomenon; it chiefly affects the trunk and face, and is one of the commonest forms of the so-called "corona veneris" at the junction of the forehead with the scalp. Diagnosis: The diagnosis of this form of syphilitic skin lesion is often difficult, apart from a good history of previous affections. The accounts given in the text-books do not give sufficient importance to the resemblance which this lesion has to tinea circinata. Many cases of late secondary syphilis, both under treatment and without it, develop a circinate eruption which at first sight closely resembles tinea circinata. The differences consist in (1) the colour of the syphilitic lesion, which, however, is often not typical; (2) the absence of the clear centre with spreading edge, which is more typical of tinea. It is also difficult to diagnose from eczema palmaris when it occurs in the palm of the hand, but differs from this by beginning in the centre of the palm.

**Vesicular and Pustular Eruptions.**—Syphilitic eczema is a rare affection. When it occurs it is of slow progress and characterised by the absence of itching, the pigmentation of the skin, the absence of weeping, and the usual association with other skin lesions of a different type. Practically, the only vesicular and pustular eruptions likely to be confounded with syphilis are varicella and variola. Sometimes, varicella occurring in an adolescent or in an adult may suggest syphilis, especially as in such cases the eruption may occur on the penis (as was the case in one case I have seen) thereby suggesting primary venereal sores, especially when such become irritated. As regards small-pox, the only likely mistake to be made is one of mild variola in a patient who has been vaccinated when a child. However, the uniform pustular eruption and the affection of face together with the constitutional symptoms should clear up the diagnosis. A pure pustular syphilide is one of the rarest of syphilitic affections excepting the ecthyma occurring in cachexia. The same remarks will apply to varicella (and it may be noted here that varicella occurring in an adult is sometimes almost impossible to diagnose from a mild variola occurring in a patient who has been vaccinated once). In fact, an eruption resembling either of these diseases should never be diagnosed as syphilis when the eruption is uniform, and there are no other absolutely definite signs of syphilis.

(*To be continued.*)

